# An In Vitro Study of the Photodynamic Effectiveness of GO-Ag Nanocomposites against Human Breast Cancer Cells

**DOI:** 10.3390/nano7110401

**Published:** 2017-11-21

**Authors:** Fozia Shaheen, Muhammad Hammad Aziz, Muhammad Fakhar-e-Alam, Muhammad Atif, Mahvish Fatima, Riaz Ahmad, Atif Hanif, Saqib Anwar, Fatima Zafar, Ghazanfar Abbas, Syed Mansoor Ali, Mukhtar Ahmed

**Affiliations:** 1 Department of Physics, Government College (GC) University, Lahore 54000, Pakistan; scarletfozia@yahoo.co.in or foziashaheen@gcu.edu.pk; 2 Department of Physics, COMSATS Institute of Information and Technology, Lahore 54000, Pakistan; drghazanfarabbas@ciitlahore.edu.pk (G.A.); mukhtarahmad@ciitlahore.edu.pk (M.A.); 3 Department of Physics, Government College (GC) University, Faisalabad 38000, Pakistan; fakharphy@gmail.com; 4 Institute of Fundamental and Frontier Science, University of Electronic Science and Technology of China, Chengdu 610054, China; 5 Department of Physics and Astronomy, College of Science, King Saud University, Riyadh 11451, Saudi Arabia; atifhull@gmail.com (M.A.); mansoor_phys@yahoo.com (S.M.A.); 6 National Institute of Laser and Optronics, Nilore 45650, Islamabad; 7 Department of Physics, University of Lahore, Lahore 54000, Pakistan; mahvish.fatima@phys.uol.edu.pk; 8 The Centre for Advanced Studies in Physics (CASP), Government College (GC) University, Church Road, Lahore 54000, Pakistan; 9 Botany and Microbiology Department, Faculty of Science, King Saud University, Riyadh 11451, Saudi Arabia; ahchaudhry@ksu.edu.sa; 10 Industrial Engineering Department, College of Engineering, King Saud University, P.O. Box 800, Riyadh 11421, Saudi Arabia; sanwar@ksu.edu.sa; 11 Department of Chemistry, GC University, Lahore 54000, Pakistan; fatimazafar09@gmail.com

**Keywords:** graphene oxide (GO), photodynamic therapy, cytotoxicity, biocompatibility, reactive oxygen species (ROS)

## Abstract

Graphene-based materials have garnered significant attention because of their versatile bioapplications and extraordinary properties. Graphene oxide (GO) is an extremely oxidized form of graphene accompanied by the functional groups of oxygen on its surface. GO is an outstanding platform on which to pacify silver nanoparticles (Ag NPs), which gives rise to the graphene oxide-silver nanoparticle (GO-Ag) nanocomposite. In this experimental study, the toxicity of graphene oxide-silver (GO-Ag) nanocomposites was assessed in an in vitro human breast cancer model to optimize the parameters of photodynamic therapy. GO-Ag was prepared using the hydrothermal method, and characterization was done by X-ray diffraction, field-emission scanning electron microscope (FE-SEM), transmission Electron Microscopy (TEM), energy dispersive X-rays Analysis (EDAX), atomic force microscopy and ultraviolet-visible spectroscopy. The experiments were done both with laser exposure, as well as in darkness, to examine the phototoxicity and cytotoxicity of the nanocomposites. The cytotoxicity of the GO-Ag was confirmed via a methyl-thiazole-tetrazolium (MTT) assay and intracellular reactive oxygen species production analysis. The phototoxic effect explored the dose-dependent decrease in the cell viability, as well as provoked cell death via apoptosis. An enormously significant escalation of ^1^O_2_ in the samples when exposed to daylight was perceived. Statistical analysis was performed on the experimental results to confirm the worth and clarity of the results, with *p*-values < 0.05 selected as significant. These outcomes suggest that GO-Ag nanocomposites could serve as potential candidates for targeted breast cancer therapy.

## 1. Introduction

Graphene has been making a noteworthy effect and shows the potential application in biomedicine, for instance anticancer, antibacterial activity, cell science and bio-distinguishing and likewise showing drug conveyance limits [1,2,3,4,5]. Graphene has fantastic properties including a high electrical and thermal conductivity, surface area and mechanical properties. Graphene oxide (GO) nanosheets can be utilized as nanocarriers for drug delivery and intracellular fluorescent nanoprobe [2,4,5]. Subsequent advancement of covalently- and noncovalently-functionalized graphene-based materials enhanced their cytotoxicity and diminished their toxicity in the physiological environment [6]. The strong penetration capabilities of graphene-based nanocomposites make them able to produce trivial adverse effects on different cellular models, describing the importance of these nanocomposites for the studies of biological effects [7,8,9,10,11].

Graphene also signifies an esteemed platform for the progress of nanocomposites, permitting the transformation of nanomaterials with diverse properties to contribute to innovative materials with enriched or advanced functionalities. Graphene toxicity has been measured by numerous research groups owing to its divergent physicochemical features such as purity, the size of the sheets and the oxidation state, which may affect its cellular uptake and biodegradation [12,13]. However, graphene can spontaneously enter the plasma membrane, and it can be easily localized in the cytosol, synchronizing with cellular organelles. Graphene produces an increase of oxidative stress and metabolic activity related to the repairing mechanisms inside cells [12,13,14].

GO is the most common member of the graphene family in the study of in vitro toxicity [10,11]. Previous studies showed that at a lower concentration, treatment with GO in a human neuroblastoma SH-SY5Y cell line caused no significant cytotoxicity. Graphene oxide produced considerable cytotoxicity with continuous exposure to the SH-SY5Y cell line at a higher concentration for time duration of 96 h [15]. Yang et al. reported their work on mice and showed the potential toxicity of nanographene sheets with polyethylene glycol after injection in mice [16]. GO has the ability to adsorb protein, such that it can yield concentration-dependent cytotoxicity, which may possibly be lessened by incubation with 10% fetal bovine serum (FBS), owing to GO’s enormously high protein adsorption ability [17].

Until now, metal nanostructures (Ag, Ni, Au, Cu) have received more attention in the medical field due to their impending influence on the environment and human health [18,19,20]. Silver nanoparticles (Ag NPs) play the vital role of a biocidal agent, and their toxicity phenomena are associated with oxidative stress and cell membrane damage [18,21]. Furthermore, silver dysregulates the mitochondrial respiratory system and decreases the effectiveness of antioxidant enzymes, such as glutathione transferases, causing the reduction of free radicals. Silver nanoparticles’ toxicity may also be related to other mechanisms, such as the inhibition of DNA synthesis, actin depolymerization, membrane instability and intracellular calcium overload, all of which induce early cell apoptosis [20,22].

Metal-incorporated graphene has generated extensive interest in various biological applications, including biosensing, photothermal therapy, as well as photodynamic therapy [23,24]. Particularly, the photodynamic process quickly produces reactive oxygen species (ROS) comprising hydroxyl radicals, superoxide ions, and singlet oxygen (^1^O_2_), with the latter associated through the main relevant agent of cellular destruction in the photodynamic practice [10,25]. Numerous methodologies have been offered to increase the effectiveness of photodynamic therapy (PDT). Occasionally, PDT adequacy was observed when nanoparticles were connected as *Photosensitizers* (*PS*) transporters, proposing that the utilization of nanoparticles can overcome the previously mentioned restrictions [14,25,26,27]. Among the different nanoparticles accessible, for instance quantum dots, nanotubes, liposomal vehicles, and gold nanoparticles, the latter has pulled in generous consideration on account of their chemical latency, superb optical properties, and nominal biological toxicity [28,29,30]. Photodynamic therapy (PDT) has been extensively studied for its high capability for medical applications, especially in the treatment of cancer. Hence, considerable attention has been given to fabricating graphene-based nanomaterials that show higher cell apoptosis death via PDT [25,30,31,32]. Therefore, in our work, we have treated a human breast cancer cell line with graphene oxide-silver (GO-Ag) nanocomposites to analyze their effectiveness using PDT. Besides, the structural and morphological characteristics of GO-Ag nanocomposites were exposed. Furthermore, mechanism of cytotoxicity GO-Ag against human breast cancerous cells was assessed. In addition, nanocomposites induced morphological changes towards MCF-7 cells which were also an integral component of the cellular mechanism relating to its therapeutic effects and cytotoxicity. The nanocomposites were considered concerning their ability to create the singlet oxygen by using chemical trapping process (DPBF; 1, 3-diphenylisobenzofuran). To potentially recommence and support earlier studies, a statistical study based on linear regression is accomplished on the experimental outcomes to help to understand the contrivance of GO-Ag on the tumor cells. Additionally, this study is also useful for the further investigation of graphene-based nanomaterials in nanomedicine and divulges the present development of photodynamic therapy via nanotechnology. 

## 2. Materials and Methods

### 2.1. GO-Ag Nanocomposites Preparation

GO was prepared by the modified Hummers method [33]. The GO (1 g) and distilled water (30 mL) were added to a 500-mL bottle and ultrasonicated for 30 min. After that, NaSH (16 g) was added with continuous stirring, and the mixture was sonicated again for 1 h at 50 °C, then the mixture was maintained under stirring for 15 h at 60 °C in order to produce the thiol group on the surface of GO. The obtained product was washed with distilled water (50 mL) and then sonicated for 20 min. Then, 0.2 M of AgNO_3_ was added to the thiol-functionalized graphene oxide (GOSH) solution under continuous stirring. The final product was obtained by centrifugation and washed with distilled water, then dried at room temperature overnight.

### 2.2. Cell Culturing (MCF-7, Breast Cancer Cell Line)

MCF-7 cells were process cultured and cultivated in a T-75 flask (Nunc, Wiesbaden, Germany) containing 10 mL of complete growth medium (Eagle’s Minimum Essential medium EMEM + 10% (*v*/*v*) FBS (fetal bovine serum) + 1% bovine insulin). Furthermore, for appropriate association, the cells were placed for incubation at 37 °C for 24 h. The MCF-7 cells (breast cancer cells) were sub-cultured twice or thrice in a week, and after, the cells were washed with 0.25% (*w*/*v*) trypsin to attain 76–80% confluence [21,34].

### 2.3. Labeling of MCF-7 Cells

MCF-7 cells were harvested at a concentration of 1 × 10^5^ cells/well in 96-well plates and incubated. Furthermore, an ascending dose of GO-Ag nanocomposites (10, 20, 40, 60, 80 and 100 µg/mL) was delivered at 37 °C for 24 h having 10% FBS and 5% CO_2_. The dose arrangement of GO-Ag with increasing concentration ranging from 10–100 μg/mL was placed in 96-well plates, while the remaining column was used as the control [34,35].

### 2.4. Photodynamic Therapy of MCF-7 Cells

For photodynamic therapy experiments, in a 96-well microplate, MCF-7 cells (breast cancer cells) were incubated with a GO-Ag concentration of 10–100 µg/mL at 37 °C. Afterward, the cells were exposed for 15 min at a Laser light of 430-nm blue wavelength, irradiated with 100 J/cm^2^. After being further cultured for another 24 h, the relative cell viabilities were then measured by the MTT assay [36].

### 2.5. In Vitro Cellular Cytotoxicity MTT Assay

Finally, to assess the cytotoxicity on MCF-7 cells, the MTT (3-(4,5-dimethyl thiazol-2yl)-2,5-diphenyl tetrazolium bromide) assay was performed. After incubation, the medium from the wells was removed. Then, 25 μL of MTT (5 mg/mL) were added into each well and again incubated for 4 h. The media was washed away again, and the cells were dispersed in 50 μL of DMSO (dimethylsulfoxide) solvent. The absorbance spectra of the samples were measured at a 595-nm wavelength by a microplate reader [37].

### 2.6. Reactive Oxygen Species Fluorescence

Intracellular ROS production was detected using the non-fluorescent compound CMH2DCFDA (Invitrogen Corporation, Carlsbad, CA, USA). The compound crosses the cell membrane and undergoes deactivation by esterases, producing the nonfluorescent CMH2DCF, which reacts with oxygen species inside the cell to produce highly fluorescent dye chloromethyl dihydro dichlorofluorescein (CMDCF). After inactivation of cells in different concentrations of GO-Ag with the recommended light dose, in humidified air with 5% of CO_2_ at 37 °C for 12 h, the cells were washed gently once in Dulbecco’s Modification of Eagle’s Medium (DMEM). Cells were loaded with 10 μM CMH2DCFDA incubated for 30 min at 37 °C and protected from light. Thereafter, cells were exposed to a light dose of 100 J/cm^2^ for 2 min to visualize for ROS production [38,39]. After delicate protocol, cells ROS fluorescence was captured with emission detection at 530 nm.

### 2.7. Characterization

The morphology of GO-Ag was ascertained by a field-emission scanning electron microscope (FE-SEM) (Nova^TM^ NanoSEM 450), transmission Electron Microscopy (JEM-1011, JEOL, Tokyo, Japan), and an atomic force microscope (AFM, Park Systems Co., Suwon, Korea). Energy dispersive X-rays Analysis (EDAX) was performed for the conformation of elemental analysis. UV-Vis spectroscopy was attained by a UV-visible spectrophotometer (Shimadzu, Kyoto Prefecture, Japan, UV-2450). X-ray diffraction (XRD) investigation of nanocomposites was achieved on a PANalytical X’Pert-PRO at room temperature and CuKα (λ = 1.54056 A). The average crystallite size was evaluated by using Scherer’s formula as mentioned below [40],

D_β_ = 0.89λ/βcosθ

where the incident wavelength of radiation is given by λ and the full width half maximum (FWHM) is given by β.

### 2.8. Cell Morphological Analysis

MCF-7 cells were plated in six-well plates (1 × 10^4^ cells per well) and incubated with the respective GO-Ag composite concentrations for 24 h. Morphological variations were observed to illustrate the disruptions induced by the GO-Ag composite in MCF-7 cells by inverted phase contrast microscopy at 20× magnification, as shown in Figure 7.

### 2.9. Statistical Analysis

The results are evaluated as the mean ± standard deviation of three independent experiments. All of the experimental data were compared using Student’s *t*-test. A *p*-value less than 0.05 was considered statistically significant using Excel software. Moreover, the parameters employed in this statistical examination using Mathematica software focused on a linear regression equation, which conveys significant evidence, such as the coefficient of determination, the intercept, the slope, the standard deviation and the variance of the slope of the regression line. To assess the performance parameters of the statistical analysis, a mean (*X*) was defined from seven independent determinations, such as the concentration of GO-Ag nanocomposites, for the obtainable data. Different statistical parameters were intended to confirm the experimental outcomes. A graph of GO-Ag alone and with PDT for cell viability and ROS with linear calibration was plotted (concentration: 10–100 µg/mL), demonstrating linearity and regression data [32].

### 2.10. Exposure of Singlet Oxygen by Chemical Trapping

1, 3-Diphenylisobenzofuran (DPBF) was employed to achieve the release of singlet oxygen (^1^O_2_) into the solution by the nanocomposites as illustrated in earlier studies [41,42]. An ethanol solution of 3 mL having 50 μM DPBF and 100 μg/mL of the GO, Ag NPs and GO-Ag or Methylene Blue (MB) solution were employed in a quartz cuvette in the dark. The experiments were performed by exposing the samples to sunshine filtered beyond a shortpass infrared filter (<550 nm). The absorbance of the solution was calculated at 410 nm, every 30 s for 3 min with a NanoDrop 2000 (Thermo Fisher Scientific, Waltham, MA, USA). Photobleaching of DPBF instigated the decreased in absorbance and noted in all experiments. The ^1^O_2_ quantum yield of the GO, Ag NPs, and GO-Ag nanocomposites in the aqueous solution were measured by using MB as a standard from the following formula:

$$\Phi_{\Delta }^{b}=\frac{\Phi_{\Delta }^{a}}{I^{a}}I^{b}$$

$\Phi_{\Delta }^{b}$ describes the ^1^O_2_ quantum yield of the nanoparticles, $\Phi_{\Delta }^{a}$ is the ^1^O_2_ quantum yield of MB that was calculated by using Rose Bengal (RB) as a standard (Φ_RB_ = 0.75 in H_2_O [43]), ‘*I*^b^’ is the slope which signifies the time for the reduction in absorption of the DPBF in the existence of the nanocomposites while ‘*I*^a^’ is the slope of MB that describes the time for the decrease in absorption of DPBF in the presence of the MB.

## 3. Results and Discussion

**Figure 1:**
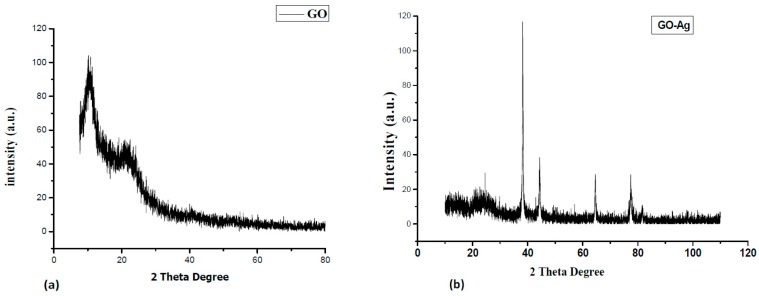
X-Ray diffraction (XRD) structure of (**a**) Graphene Oxide (GO) (**b**) Graphene Oxide-silver (GO-Ag) nanocomposites.

**Figure 2:**
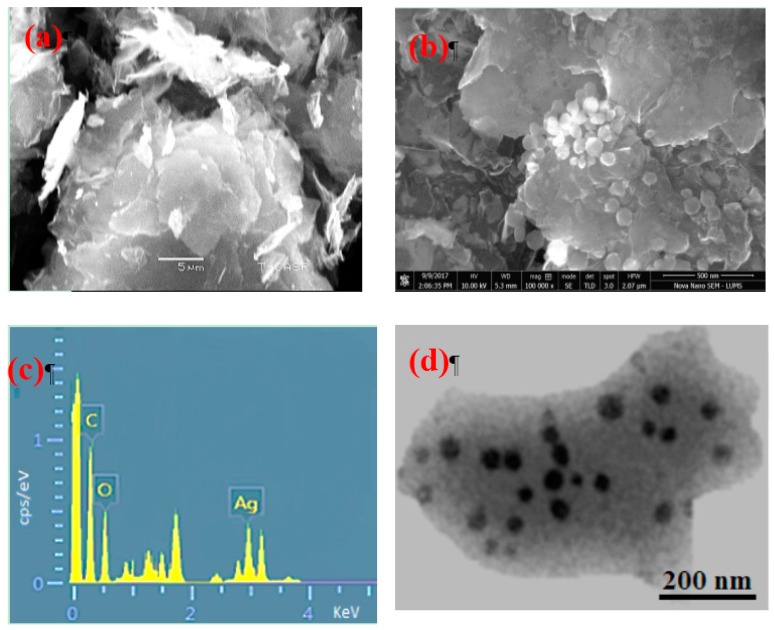
Scanning Electron Microscopy (SEM) analysis of (**a**) GO (**b**) GO-Ag nanocomposites (**c**) Energy Dispersive X-rays (EDAX) analysis of GO-Ag nanocomposites (**d**) Transmission electron microscopy (TEM) of GO-Ag.

**Figure 3:**
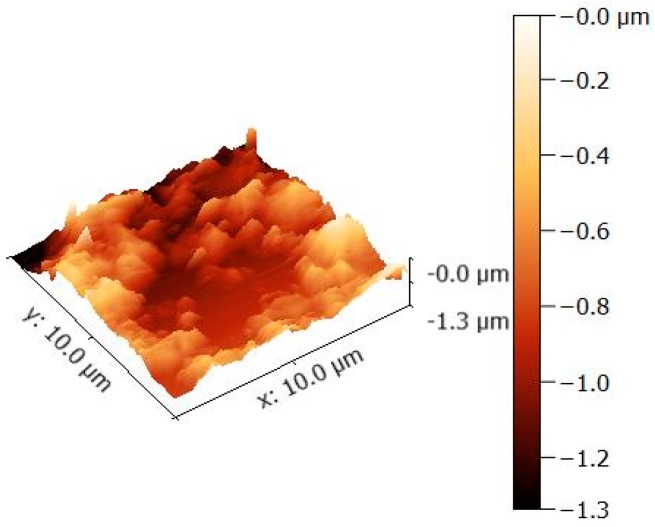
Atomic Force Microscopy (AFM) analysis of GO-Ag nanocomposites.

**Figure 4:**
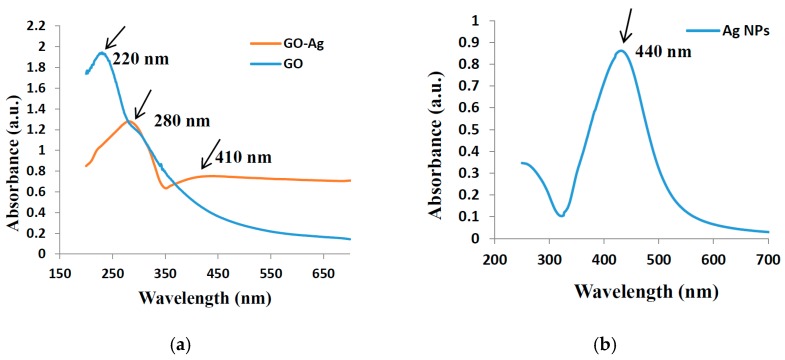
UV–visible of (**a**) GO and GO-Ag nanocomposites (**b**) Ag NPs.

Figure 1a explains the X-ray diffraction (XRD) of GO, a broad diffraction peak for GO appeared at 2θ = 10°, which is attributed to the (001) lattice plane of GO. The interlayer d-spacing corresponding to this peak was calculated 0.93 nm that acquainted the stacking between the GO nanosheets [16,17]. The XRD patterns in Figure 1b exhibited that GO incorporated with silver Nanoparticles is crystalline in nature with diffraction peaks at 38.1°, 44.4°, 64.7° and 77.4° corresponding lattice planes (111), (200), (220) and (311), respectively, of the cubic structure (JCPDS Card Number 07-0783). Moreover, the peak for GO was disappeared after decoration of Ag NPs, that can be observed in XRD spectra GO incorporated with Ag Nanoparticles. Therefore, the functionalization of the GO surface with the Ag NPs might prevent the graphene sheets from restacking [31,33]. The average crystallite size of deposited Ag nanoparticles was measured by the Scherer’s formula using dominant peak (111), determined to be about 28 nm. Scanning electron microscope (SEM) images of GO exposed a thin transparent layer of the GO sheet and a smooth surface with small wrinkles and folded edges, which is consistent with previous reports as seen in Figure 2a [21,31]. GO-Ag displayed the spherical like shinning Ag nanoparticles decorating on the GO sheets, indicating that Ag particles were successfully attached and evenly dispersed on the GO sheets, as seen in Figure 2b. The size distribution of deposited Ag particles on GO was between 30 and 35 nm, which confirms the XRD data. The composition analysis of the GO-Ag nanocomposites from the energy dispersive X-rays Analysis (EDAX) plot of the field-emission scanning electron microscope (FE-SEM) images is shown in Figure 2c. The EDAX interpretations indicated that the required phase of Ag is presented in the samples. Furthermore, The EDAX outcomes confirmed the occurrence of carbon and oxygen with Ag NPs in the GO-Ag nanocomposites. Figure 2d showed the transmission Electron Microscopy (TEM) of the GO incorporated with Ag NPs in which spherical NPs of Ag decorated on the GO sheet of the average size of 43 nm was seen, that also described the confirmation of the XRD and FE-SEM results. Figure 3 displayed the AFM image of as-prepared GO-Ag on mica. After stacking the Ag NPs, the thickness of GO-Ag increased to about 10 nm, which shows that the Ag NPs were loaded on the surface of the GO sheets, which can be observed from the cross-sectional view. The UV-Vis spectrum revealed the absorbance spectra of GO, GO-Ag NPs and Ag NPs alone as shown in Figure 4. The obtained GO exhibited a maximum absorption peak centered at 220 nm and only Ag NPs is about 440 nm. After incorporation of Ag NPs with GO, the peak of Ag NPs shifted toward the shorter wavelength, this may be due to the surface Plasmon resonance of Ag NPs. [31].

**Figure 5:**
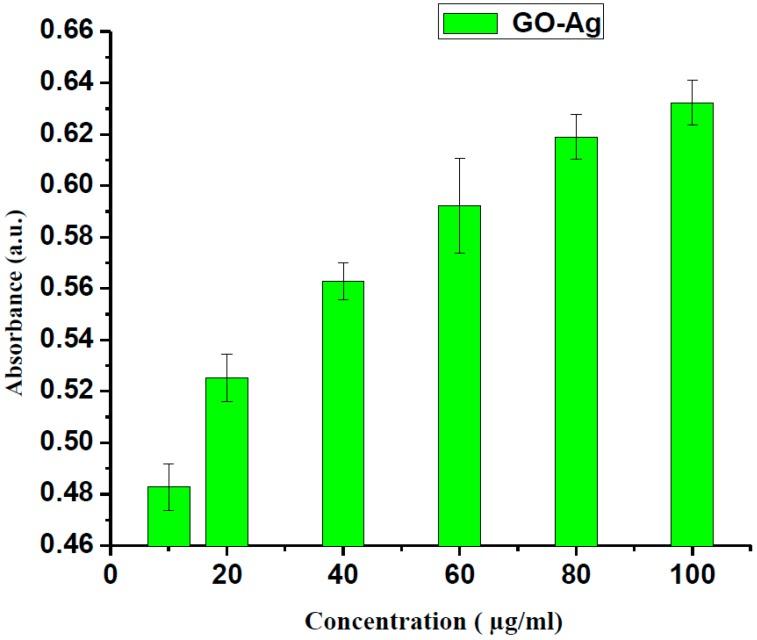
Absorbance versus concentration of GO-Ag nanocomposites.

Graphene-based materials have distinctive physicochemical properties and are functional for several prospects. However, their biological properties in organisms will finally determine their destiny in the future. In many recent studies, researchers focused on cell surface adhesion biofilms. Numerous studies have shown that the biofilm phenotype can be described in terms of the genes expressed by biofilm-associated cells. The supramolecular self-assembly approach and their hybrid/complex forms were employed with a laser irradiation mechanism [44,45,46] for the successful implementation of targeted photothermal and photodynamic efficacy. The promising biomedical uses of graphene/silver nanocomposites have been considered for numerous applications, including antibacterial properties and nanocarriers for controlled stacking on drug delivery conveyance as an anticancer agent [47,48]. An organized study was performed to assess the lethality of GO-Ag to MCF-7 cells, a generally-utilized model cell line for toxicological study. The said nano-structural material was employed to decide the likelihood of cell demise because of mechanical stress/cell death. In this work, we attempted to follow the absorbance/optical density measurement by taking different concentrations of GO-Ag as explained in Figure 5 after an uptake time of 24 h. The current experimental study evaluated the optimal density/absorbance of usefulness of GO-Ag in a breast cancer cell line. In addition, it was found that by increasing the concentration of GO-Ag, the mean absorbance/optical density of the stated NPs increased to 0.6 a.u., which is significant, as shown in Figure 5. The results showed the dependency of the significant loss of cell viability and the enormous reactive oxygen species (ROS) liberation under laser light irradiation, which depicted the high profile cancerous cell/tissue injury via cell necrosis/apoptosis, respectively. These outcomes showed that GO-Ag revealed the concentration/dose dependent cytotoxicity with MCF-7 cells.

**Figure 6:**
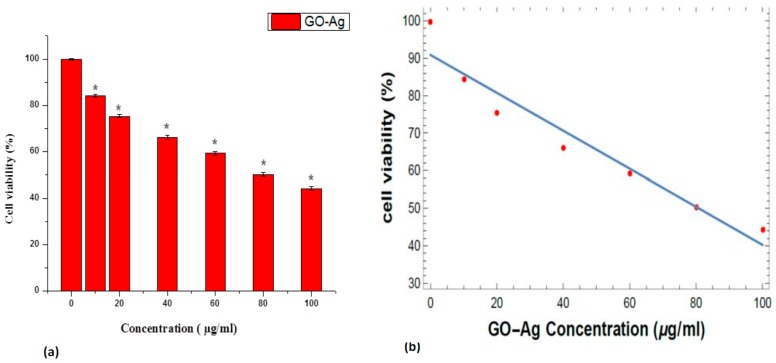
(**a**) Loss in cellular viability (%) in MCF-7 breast cell line treated with GO-Ag nanocomposites after 24 h, *t*-test (* *p* < 0.05) (**b**) Linear calibration Plot of GO-Ag vs. cell viability.

The cytotoxicity effects of GO-Ag nanocomposites were evaluated using the methyl-thiazole-tetrazolium (MTT) assay. In a dose-dependent manner [49], GO showed almost 13% retained cell viability of MCF-7 cells and cytotoxicity. The fluorinated form of graphene oxide (FGO) showed no toxicity to human breast cancerous cells [50]. The toxicity of graphene-carbon paste (GCP) at four different concentrations (1, 2.5, 5 and 10 wt. %) on MDA-MB-231 breast cancer cells also using the MTT assay was explored by Waiwijit and coworkers [51]. The impact of graphene oxide on the viability using the MTT assay was also investigated by de Marzi et al. with the same cell line. Graphene oxide was used for two different chip sizes (1.32 μm and 130 nm) and a range of concentrations (10, 50 and 100 μg/mL). After 24 h of incubation with both types of GO, the results showed insignificant reduction in the viability of the A549 cells [52]. The toxicity of GO has also been explained in the HBI.F3 human neural stem cell line and BEAS-2B human lung cells. In BEAS-2B cells, the significant concentration and temporal reduction in cell viability were observed at concentrations of 10–100 μg/mL by the MTT assay, and both early and late apoptosis of cells were improved when compared to the control [53]. HBI.F3 cell viability was decreased with increasing GO nanopellet concentration (25–200 μg/mL), which was verified by the MTT assay and differential pulse voltammetry, a microscopic imaging tool [54]. Breast cancer cells were treated via diverse concentrations (10–100 µg/mL) of GO-Ag for 24 h, as seen in Figure 6, indicating the decrease in cell viability in a dose-dependent manner. The reduction in cell viability was seen to be approximately 44% at a concentration of 100 µg/mL, as shown in Figure 6a, which is significant (*p* < 0.05, *t*-test). Liao et al. [55] established that the cytotoxicity of erythrocytes and skin fibroblasts increased with the concentration of GO. In another study, it was revealed that the cytotoxic effect on HepG2 cells increased as the concentration of GO increased [56]. However, Zhou et al. discussed that GO-Ag reduced the cellular viability of lung cancer cells and hepatocellular carcinoma cells [57]. Nevertheless, previous data have provided evidence that GO-Ag nanocomposites vindicate two simple criteria for a viable anticancer agent, i.e., tumor specificity and nominal toxicity to the normal cells. Moreover, the aforementioned accessible data explained, unambiguously, that the cellular viability loss was in a dose-dependent manner [55,56]. Therefore, the toxicity of GO-Ag to breast cancer cells may also be synergistic, which maximized the interaction among the cells. The toxicity related to the synergistic consequence of GO-Ag nanocomposites with respect to breast cancer cells can include disruption of cell wall/membrane and the generation of oxidative stress [58]. In addition, the linearity represents the value of the regression equation analysis (*Y* = 90.8638 − 0.5059*X*) from the calibration data attained (*n* = 7) using GO-Ag cell viability vs. concentration in Figure 6b. The intercept had a value of 90.8638 with a negative slope of 0.505881, and the standard error for the slope in regression (Std err = 7.3858) was significant. The other parameters included the standard deviation (SD = 19.54), the correlation coefficient (*r*^2^ = 0.9385) with a *p*-value = 4.46 × 10^−5^ and *t*-statistics value (*t*_s_ = 9.2692) and *t*-critical value (*t*_crit_ = 1.943) showing the significance of results as *t*_crit_ < *t*_s_. Hence statistical results corroborated the accuracy of the experimental data.

**Figure 7:**
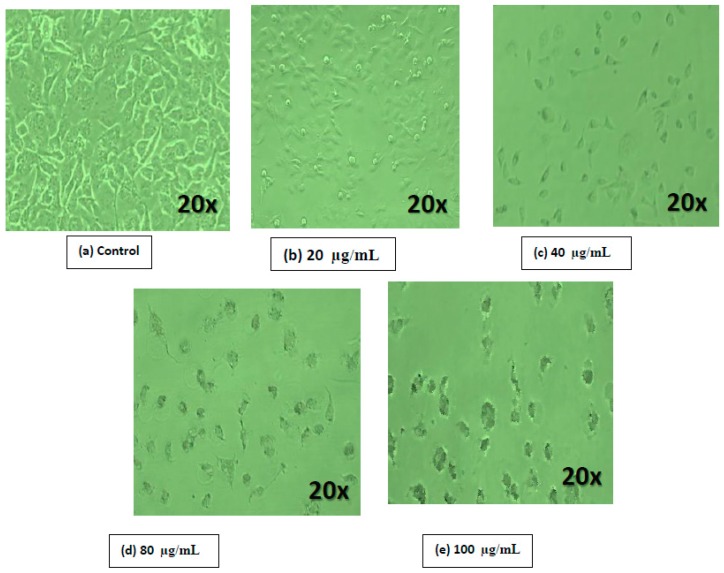
Morphological changes of breast cells when treated with GO-Ag. (**a**) control; (**b**) 20 µg/mL; (**c**) 40 µg/mL; (**d**) 80 µg/mL; (**e**) 100 µg/mL.

Previous studies showed that the use of graphene-related derivatives induced apoptosis in cancerous cells. Therefore, we examined whether the addition of the GO-Ag nanocomposite to MCF-7 cultures produced any pronounced effect on the cellular morphology [58]. In addition, to support the results of the cell viability assay, we further evaluated the effect of the GO-Ag nanocomposite on the cell morphology of breast cancer cells. Figure 7 shows a photomicrograph composite of the MCF-7 cells incubated for 24 h in the presence or absence (control) of GO-Ag nanocomposites. The control MCF-7 cells were appeared as large adherent cells, epithelial and having long arms, with indistinct cell borders. MCF-7 cells when treated with GO-Ag looked different from those of the control group. A reduced number of cells and a significant effect on the cell morphology were observed at different concentrations of GO-Ag, such as 20, 40, 80 and 100 μg/mL, as seen in Figure 7b–e respectively in the breast cancer cells. At higher concentrations, treated cells appeared as significantly less dense with shrunken arms. Similarly, Jaworski et al. [59] stated the variable toxicity of GO and rGO in glioma cells. In another study, an abridged amount of cells and a noteworthy outcome on the cell morphology were perceived in A2780 cells treated with GO. The GO-treated cells appeared slightly dissimilar from those of the control group [58].

**Figure 8:**
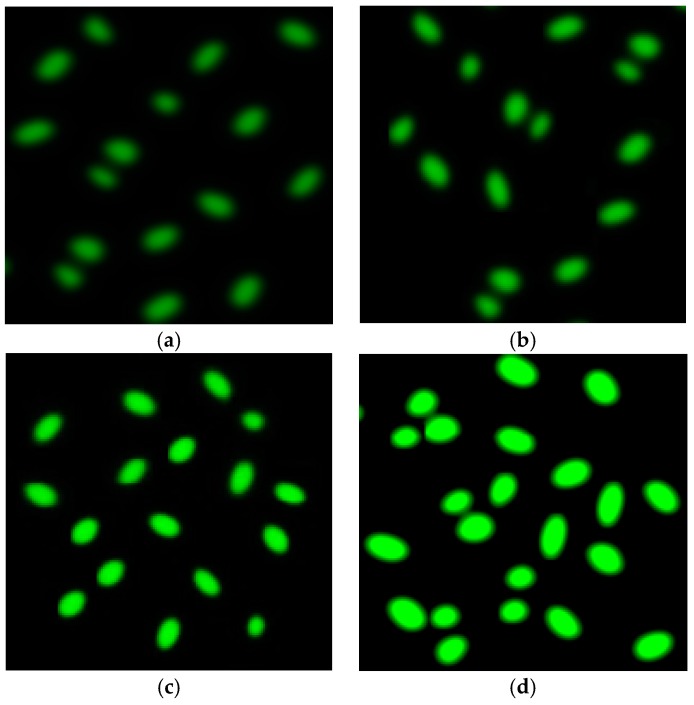
Qualitative characterization of reactive oxygen species (ROS) formation by H2DCFDA staining using fluorescence microscopy (**a**) Control MCF-7 cell line; (**b**) MCF-7 cells treated with GO; (**c**) MCF-7 cells treated with Ag NPs; and (**d**) MCF-7 cells treated with GO-Ag nanocomposite.

Several studies have reported the importance of ROS in cytotoxicity. ROS is one of the proposed toxicological mechanisms of various nanomaterials, including graphene. ROS accumulation is one of the mechanisms for the cell killing effect (cell apoptosis/cell necrosis) [57,58,59]. In addition, ROS targets the mitochondria, which leads to cell apoptosis (cell death) via vascular blockade [58,59]. ROS has the ability to create the oxidative stress that damages the cellular fragments such as cell membranes, DNA, and cellular proteins, which may lead to cell death [50,55,56]. Hydroxyl radical (OH·) is one of the ultimate reactive oxygen radicals that responds quickly through natural molecules originated in viable cells, particularly, lesser density lipoproteins receptors are in majority in the cancerous cells. The incidence of the intracellular ROS was determined by using 2′,7′-dichlorodihydrofluorescein diacetate (H2DCFDA) staining. H2DCFDA is well known cell-permeate indicator for ROS [60]. It is non-fluorescent dye until the acetate groups are removed by intracellular esterase and oxidation occurs within the cell, resulting in a reduced intermediate that can subsequently be oxidized in the presence of ROS and thus fluoresces. Figure 8a–d showed the intracellular ROS generation by fluorescence microscopy image of MCF-7cells. It is perceived that the green fluorescence depicted the presence of intracellular ROS in the cells. Visual effect of (a) control MCF-7 cells (b) GO exposed (c) Ag exposed and (d) GO-Ag exposed to MCF-7 cells were employed. Production of ROS and loss in cellular viability demonstrated the excellent agreement relation towards cell killing effects. These effects/results conclude that GO-Ag interacts with the tissue fluorophores and leads to ROS production resulted cell killing remedy. In addition as long as concentration of GO-Ag increased, ROS fluorescence visualization was also increased. GO-Ag corresponding ROS is dominant over rest of the sample like Ag NPs and GO individually towards tissue. From Figure 8d, it is cleared that MCF-7 cells when treated with GO-Ag are very dominant with significant intensity of fluorescence as compared to GO and Ag NPs alone as seen in Figure 8b,c. 

**Figure 9:**
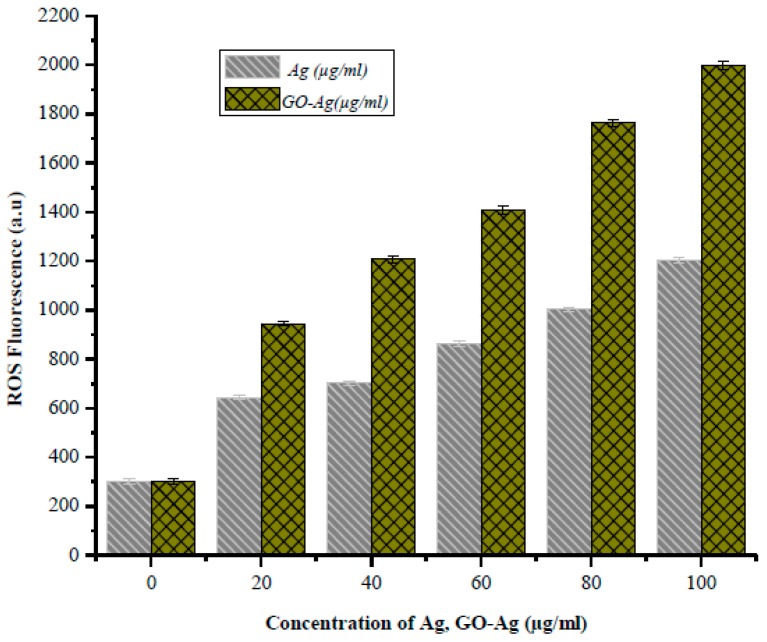
ROS Fluorescence in MCF-7 cells Model after Labeling with Ag NPs and GO-Ag (microgram/mL).

In Figure 9, depicted liberation of ROS is directly proportional to Ag alone and GO-Ag accumulation into biological model, but GO-Ag is more dominant as compare to individual Ag NPs when exposed to the biological cells. These results resembled with perfect agreement with previous published data [61,62,63], as longer as concentration increased, fluorescence Intensity were also increased. In addition, it is confirmed via manifold techniques that release of singlet oxygen (^1^O_2_) into the solution by the nanoparticles as described previously [57,58,59]. It was investigated that GO-Ag produced more oxygen stress in the tumorous targeted site due to less metabolic activity as compare to healthy part, in healthy tissue their enzymes stability and routine metabolic activity their immune system act as scavenging tool towards this new developed anticancer agent. Basically oxidative stress can cause of cell killing effect via damage proteins, DNA, and lipids, and is involved which precursor to many diseases. Damage to infected cells caused by oxidative stress is related to increased levels of ROS. During oxidative stress, hydrogen peroxide levels are often increased and catalase levels are decreased inside cells [53,62].

**Figure 10:**
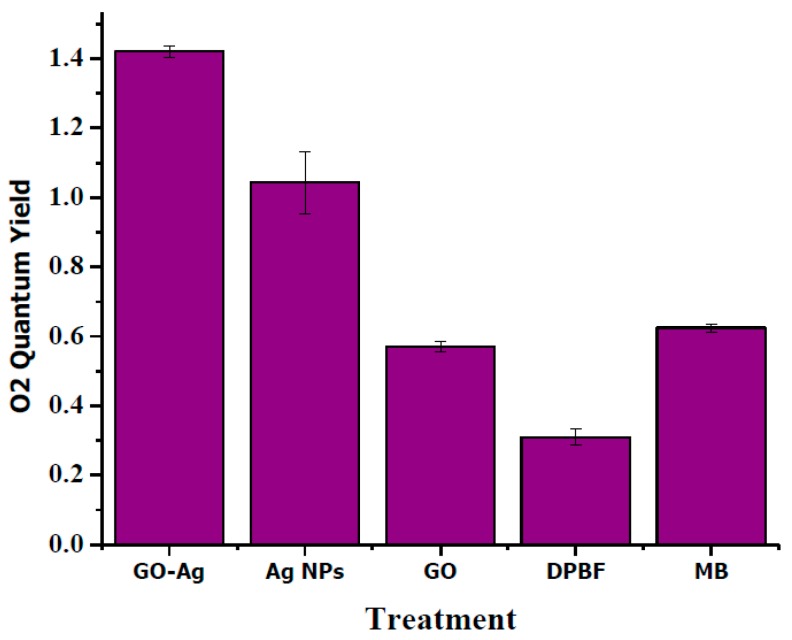
Quantum yield of singlet oxygen of GO-Ag, silver nanoparticles (Ag NPs), GO in comparison to MB.

The release of ^1^O_2_ into aqueous solution was estimated indirectly by using the DPBF assay. DPBF reacts irreversibly with ^1^O_2_ causing a decrease in its absorption intensity at 410 nm. The different NPs (100 µg/mL) were mixed in DPBF solution and upon irradiation, absorption was measured over a period of time [41,42]. As shown in Figure 10, Quantum yield of only DPBF had a slight increase in the ^1^O_2_ production but methylene Blue produced a significant escalation in the ^1^O_2_ levels. Graphene Oxide too contributed to the significant increase in the ^1^O_2_ levels but its level was marginally lesser than that of MB. Silver NPs produced the similar induction of ^1^O_2_ that was significantly higher than graphene oxide alone. For GO-Ag nanocomposites a considerable stronger induction in ^1^O_2_ production was observed.

**Figure 11:**
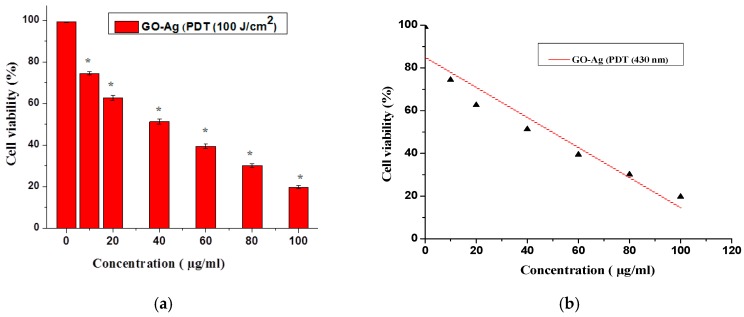
(**a**) Cellular viability of GO-Ag treated breast cell carcinoma using PDT (100 J/cm^2^), *t*-test (* *p* < 0.05); (**b**) Linear calibration Plot of GO-Ag vs. cell viability under PDT (100 J/cm^2^).

The PDT technique for cancer detection depended on suitable parameters, e.g., nanoparticle optimal dose and producing the photochemical reaction after irradiation of the proper wavelength of light, which led to cell death. In this study, the possible uptake of GO-Ag was used to trace the photodynamic effects when exposed with a light dose, as shown in Figure 11a. The optimum laser dose of 100 J/cm^2^ was selected to check the phototoxicity of GO-Ag concentrations from 10–100 µg/mL, using laser light of blue wavelength (430-nm).Cellular loss up to 20% was seen and is statistically significant (*p* < 0.05, *t*-test). In comparison of Figure 6a and Figure 11a, a significant reduction of cell viability of about 25% towards MCF-7 cells was observed, which was more under laser irradiation than GO-Ag alone. For the statistical analysis of the results, in Figure 11b, the percent of cell viability after treatment with GO-Ag concentrations is plotted under light irradiation of 100 J/cm^2^, showing the linearity using regression equation analysis (*Y* = 97.8666 − 0.857*X*) of the calibration data (*n* = 7). The intercept had a value of 97.8666 with a negative slope of 0.857, and the standard error for the slope in regression is (Std err = 0.08743). The other parameters included the standard deviation (SD = 27.39155), the correlation coefficient (*r*^2^ = 0.9406) with a *p*-value = 0.00152 and *t*-statistics value (*t*_s_ = 6.3251) and t-critical value (*t*_crit_ = 1.6354) showing the significance of results as *t*_cri_ < *t*_s_. Hence statistical results corroborated the accuracy of the experimental data. In recent studies, it was shown that photodynamic therapy can generate ROS and produce mitochondrial destruction, which led to apoptotic/necrosis [64,65]. Hybrid GO/TiO2 induced the apoptotic response and had excellent photodynamic activity [66] when exposed to a light dose of 48.6 J/cm^2^. In another study, a laser of a 671-nm wavelength reduced the cellular viability using photosensitizer chlorin e6 (Ce6) and rGO-PVP-Ce6 at diverse concentrations for 24 h at room temperature [21].

**Figure 12:**
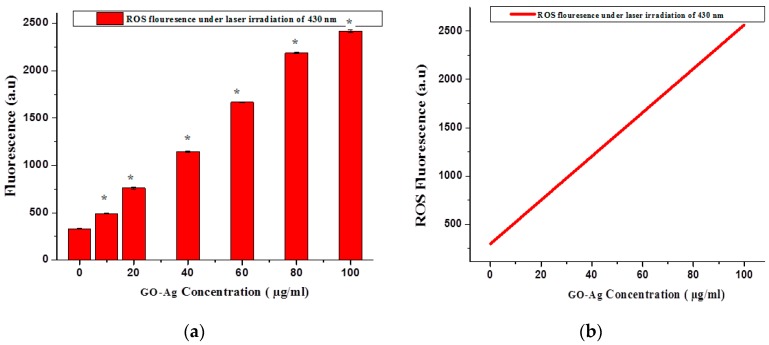
(**a**) ROS generation under light irradiation of suitable wavelength (430 nm), *t*-test (* *p* < 0.05); (**b**) Linear calibration Plot of GO-Ag concentrationvs. ROS fluorescence under PDT (100 J/cm^2^).

Kolarova et al. demonstrated that ROS overproduction can cause severe cell damage, which leads to cell death, and they made this statement after the investigation of the synergistic mechanism between sonodynamic therapy (SDT) and PDT by using MCF-7cells as an in vitro model [67]. To investigate the effect of the GO-Ag nanocomposite of different concentrations (10–100 µg/mL) on ROS generation under light irradiation of 430 nm, the results clearly indicated that GO-Ag profoundly exaggerated ROS generation when compared to control. It is clearly observed in Figure 12a, a significant rise in the fluorescence of ROS production was observed under laser irradiation parameters and is statistically significant (*p* < 0.05, *t*-test). The statistical analysis for ROS generation after treatment with GO-Ag concentrations was plotted under light irradiation of 100 J/cm^2^, showing the linearity using regression equation analysis (*Y* = 342.65 + 19.677*X*) of the calibration data (*n* = 7) as seen in Figure 12b. The intercept had a value of 342.65 with a positive slope of 19.677, and the standard error for the slope in regression is (Std err = 278.4517). The other parameters included the standard deviation (SD = 662.4739), the correlation coefficient (*r*^2^ = 0.9357) with a *p*-value = 0.0025 and *t*-statistics value (*t*_s_ = 4.2764) and *t*-critical value (*t*_crit_ = 1.942) showing the significance of results as *t*_crit_ < *t*_s_. Hence statistical results corroborated the accuracy of the experimental data.

**Figure 13:**
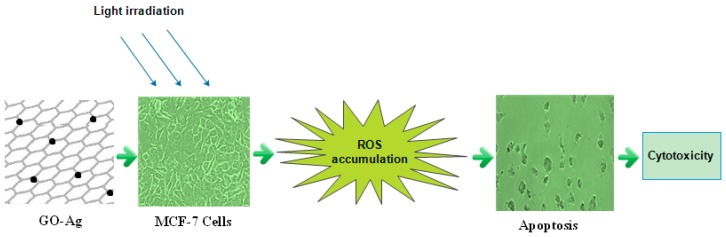
Schematic diagram represented the ROS accumulation by GO-Ag under light irradiation of 430 nm.

The present study exhibited that GO-Ag under a suitable wavelength of light exposure not only liberated reactive oxygen species (ROS), but also arrested the cell cycle by the inhibition MCF-7 cells proliferation through encouraging oxidative stress, which would be valuable for developing highly operative graphene-related aggregates for use in biomedicine [58,63,64]. Its schematic diagram is presented below in Figure 13.

In summary, this study evaluated the cytotoxicity and photodynamic effectiveness of GO-Ag nanocomposites towards MCF cells. The reduction of cell viability and the ROS induced by the GO-Ag under light irradiation suggested a synergistic effect. To the best of our knowledge, this is the first report concerning the phototoxicity of graphene oxide-silver nanocomposites to breast cancer cells that also examined the nanocomposite’s permanence. Despite the uniqueness of this work, further effort is still required to improve knowledge of the phototoxicity mechanisms produced by graphene-metal-based nanocomposites.

## 4. Conclusions

In this experimental study, GO-Ag was prepared using a hydrothermal method and characterized by applying diverse techniques, such as AFM, SEM, XRD analysis and UV-Vis spectroscopy. Cellular uptake measurements and cytotoxic evaluations were performed using an MTT assay, cell morphological analysis and laser exposure (100 J/cm^2^). Cellular loss was also confirmed by inverted microscopy at various concentrations that showed that GO-Ag produced significant toxic effects in MCF-7. GO-Ag exhibited significant cytotoxic differences after the laser exposure and revealed apoptotic activity against a breast cancer cell line. Significant loss of cell viability (up to 65% under light exposure) exposed the dependency of ROS liberation and prominent cancer cell/tissue damage via cell necrosis/apoptosis (by inducing oxidative stress). The combined effect of GO-Ag with photodynamic therapy in our study generated a synergistic outcome, which revealed that the combined process effectiveness was higher than the individual efficacies of the nanocomposites. To potentially recommence and support earlier studies, a statistical study based on linear regression was accomplished on the experimental outcomes to help to understand the effect of GO-Ag nanocomposites on the tumor cells. This considerable photodynamic effect of similar graphene-based photosensitizer encourages future investigations, which could be applied for biomedical applications, specifically cancer therapy. However, advance investigations are essential to clarify the accurate mechanism(s) of graphene-based photosensitizer in delivering their cargo, and to assess them in cancer therapy in vivo.
